# Acute Myocardial Response to Stretch: What We (don't) Know

**DOI:** 10.3389/fphys.2015.00408

**Published:** 2016-01-05

**Authors:** João S. Neves, André M. Leite-Moreira, Manuel Neiva-Sousa, João Almeida-Coelho, Ricardo Castro-Ferreira, Adelino F. Leite-Moreira

**Affiliations:** Department of Physiology and Cardiothoracic Surgery, Faculty of Medicine of the University of PortoPorto, Portugal

**Keywords:** cardiac function, frank starling mechanism, myocardial stretch, neurohumoral adaptation, slow force response

## Abstract

Myocardial stretch, as result of acute hemodynamic overload, is one of the most frequent challenges to the heart and the ability of the heart to intrinsically adapt to it is essential to prevent circulatory congestion. In this review, we highlight the historical background, the currently known mechanisms, as well as the gaps in the understanding of this physiological response. The systolic adaptation to stretch is well-known for over 100 years, being dependent on an immediate increase in contractility—known as the Frank-Starling mechanism—and a further progressive increase—the slow force response. On the other hand, its diastolic counterpart remains largely unstudied. Mechanosensors are structures capable of perceiving mechanical signals and activating pathways that allow their transduction into biochemical responses. Although the connection between these structures and stretch activated pathways remains elusive, we emphasize those most likely responsible for the initiation of the acute response. Calcium-dependent pathways, including angiotensin- and endothelin-related pathways; and cGMP-dependent pathways, comprising the effects of nitric oxide and cardiac natriuretic hormones, embody downstream signaling. The ischemic setting, a paradigmatic situation of acute hemodynamic overload, is also touched upon. Despite the relevant knowledge accumulated, there is much that we still do not know. The quest for further understanding the myocardial response to acute stretch may provide new insights, not only in its physiological importance, but also in the prevention and treatment of cardiovascular diseases.

## Introduction

The heart has a central role in the maintenance of cardiovascular homeostasis, which requires the ability to continuously adapt its function to different hemodynamic conditions.

**Figure 1:**
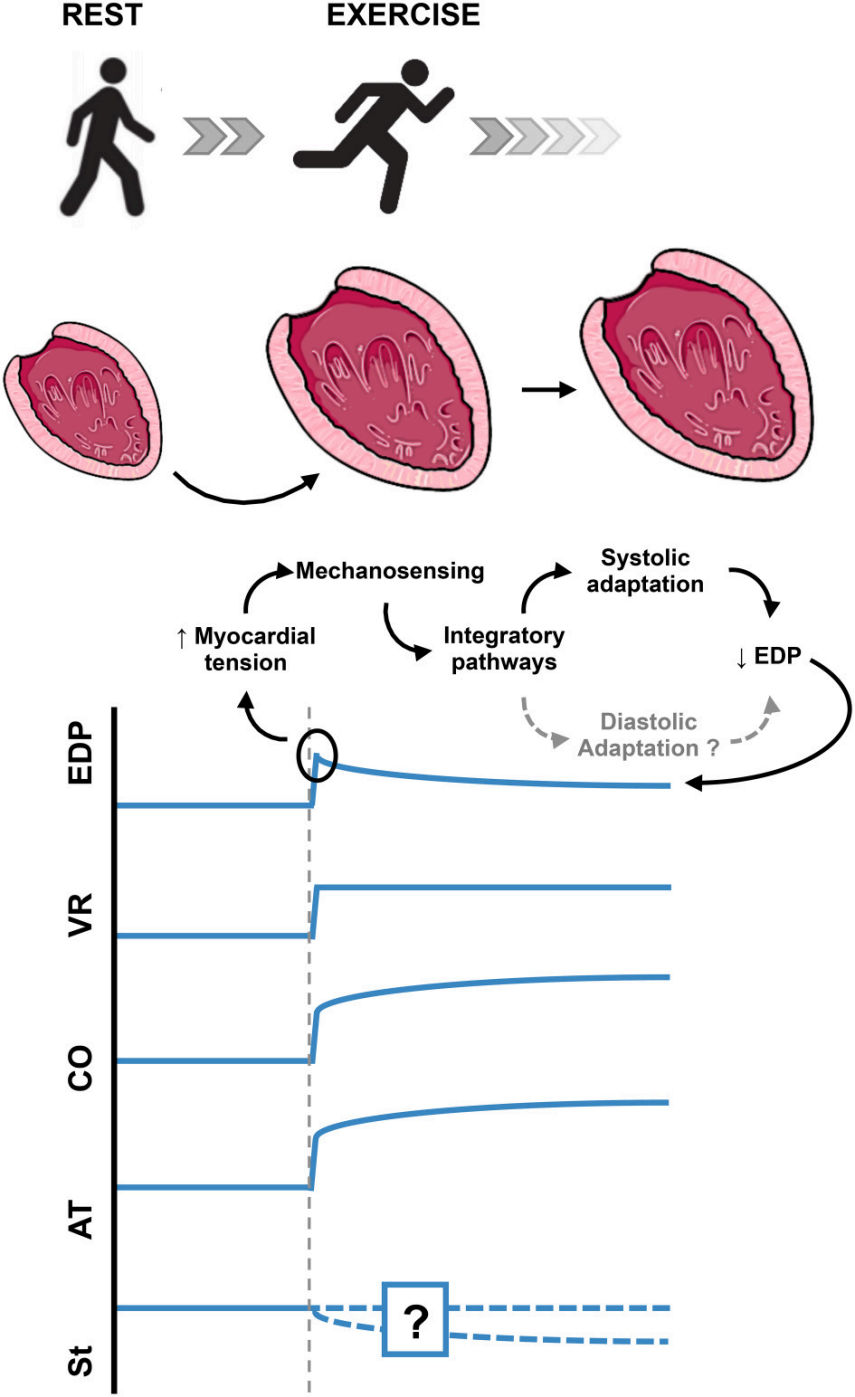
**Acute cardiac overload**. Schematic example of the normal intrinsic adaptation of the left ventricle to acute increase in preload. In this example, the additional modulation by autonomic nervous system is ignored, in order to highlight the intrinsic myocardial response to acute stretch. Myocardial stiffness changes in response to acute myocardial stretch have not yet been adequately evaluated. EDP, End Diastolic Pressure; VR, Venous Return; CO, Cardiac Output; AT, Active Tension; St, Myocardial Stiffness.

One of the most frequent challenges to myocardium is acute stretch, as result of acute hemodynamic overload. Acute myocardial stretch can be observed in various physiological and pathophysiological conditions (e.g., exercise, myocardial ischemia, hypertensive crises, valvular diseases, and heart failure). For example, at the start of aerobic exercise (Figure 1), the pumping action of skeletal muscle contraction increases the venous return, leading to cardiac chamber dilation and acute myocardial stretch (Nóbrega et al., 1995). This increase in end-diastolic volume leads to an increase in end diastolic pressure that, in the absence of adequate response, would lead to pulmonary and systemic congestion.

Being such a common challenge, one can expect the heart to have adequate intrinsic physiologic mechanisms to respond to this stimulus. The adequate physiological response to the acute increase in end-diastolic pressure must include: activation of stretch sensing molecules (the sensing organ), intermediary mechanisms (afferent, integrator and efferent pathways), and effector mechanisms that would ultimately allow the heart to reduce its increased end-diastolic pressure (Figure 1). This could, in theory, be achieved by two different mechanisms:

- A systolic adaptation: an increase in contractility, leading to an increased ejection volume and, consequently, a decreased end diastolic volume.
- A diastolic adaptation: an increase in cardiac compliance, which would allow accommodating more blood at inferior end diastolic pressures.

Both adaptations would increase cardiac output, which would also be appropriate to offset the increase in venous return.

The first described mechanism of cardiac adaptation to an acute hemodynamic overload has been known for about a century (Katz, 2002). An increase in either venous return or aortic resistance leads to an increased end-diastolic volume and to an immediate increase in contractility and stroke volume. This response is presently known as Frank-Starling Mechanism (FSM) due to the contribution of Ernest H. Starling and Otto Frank to its description (Patterson and Starling, 1914; Frank, 1959) and given its relevance for the cardiovascular homeostasis it is also known as the “law of the heart.” This response is mainly attributed to enhanced myofilamental responsiveness to Ca^2+^. There is a direct proportionality between sarcomere length and the sensitivity of the sarcomere to Ca^2+^, such that more force is generated at a given concentration of Ca^2+^ as sarcomere length is increased (de Tombe, 2003). Several mechanisms have been proposed for this myofilament length dependent activation: the degree of thin and thick myofilaments overlap may determine the potential availability of cross-bridges (Endoh, 2008); the reduction of the distance between the thick and thin filaments with the elongation of the sarcomere may contribute to the approximation of the myosin heads to actin increasing the probability of strong cross-bridge formation (Irving et al., 2000) or the increased titin's passive tension may potentiate the binding between the actin and the myosin filaments, contributing to an increase in the contraction force (Fukuda and Granzier, 2005). More recently, it was proposed by de Tombe et al. that a length modulated regulation of thin filament activation state, rather than a more direct mechanism involving myosin–actin affinity, mediates the myofilament length dependent activation (de Tombe et al., 2010).

In 1912, von Anrep described that, after the initial change in muscle length and contractility induced by clamping the heart outflow, it is observed a progressive and time-dependent increase in force development that goes beyond the force immediately achieved after stretch and is responsible for the return of end-diastolic volume back to its original value (Von Anrep, 1912). This second progressive increase in force development, was demonstrated *in vitro* by Parmley and Chuck in 1973, being since then synonymously called slow force response (SFR; Parmley and Chuck, 1973). Contrarily to the FSM, the main mechanism responsible for the SFR appears to be a progressive increase in Ca^2+^ transient (Alvarez et al., 1999).

The myocardial response to stretch is dependent on the phosphorylation status of cardiomyocytes proteins. Several signaling pathways are known to induce phosphorylation or dephosphorylation of sarcomeric proteins and ion channels. Phosphorylation of troponin I (TnI) results in reduction of myofilament Ca^2+^ sensitivity and an increase in crossbridge cycling rate, leading to acceleration of relaxation and an increased contractility (Layland et al., 2005). Myosin binding protein C (MyBP-C) phosphorylation increases actin–myosin crossbridge kinetics, also enhancing relaxation and contraction (Mamidi et al., 2014). On the other hand, titin phosphorylation is associated with a modulation of its passive tension (and consequently cardiomyocyte passive tension), either decreasing or increasing it, according to the site of phosphorylation (Castro-Ferreira et al., 2011). Phosphorylation of L-Type Ca^2+^ channel and SR Ca^2+^ release channel (ryanodine receptor, RyR2) are associated with an increased Ca^2+^ transient improving the contractile function (Berridge et al., 2003). Phospholamban (PLB) is another protein frequently targeted for phosphorylation; its phosphorylation disinhibits the sarco/endoplasmic reticulum Ca^2+^ ATPase (SERCA) increasing the uptake of cytoplasmic Ca^2+^ which improves both relaxation (by hastening the decrease in free Ca^2+^ concentration) and contraction (by increasing the Ca^2+^ available for release by the sarcoplasmic reticulum; Berridge et al., 2003).

Both FSM and SFR highlight a highly effective systolic adaptation to an acute hemodynamic overload. A diastolic adaptation *per se*, although theoretically appropriate to the underlying challenge, remains largely unknown. Until this moment, no single study directly evaluated the diastolic response to an acute myocardial stretch. Throughout this review, we will emphasize the mechanisms that suggest that a simultaneous diastolic adaptation is highly probable.

In this paper, about 100 years after the initial publication of E. Starling about the relevance of stretch to heart function, we intend to review the great progress in understanding of the myocardial adaptation to acute stretch, as well as highlight the gaps in the understanding of this mechanism.

### Myocardial mechanosensing

Mechanosensors are structures capable of perceiving mechanical signals and activating pathways that allow transduction of the signal into biochemical responses. The external forces exerted on the myocardium are transmitted through the sarcolemma (and associated adhesion structures: intercalated disks and costameres), cardiomyocyte cytoskeleton, and sarcomeric proteins. Concurrently, the sarcomeres generate forces that propagate in the opposite direction. Several mechanosensor molecules in this mechanical chain have been identified, being the best characterized ones located in Z-line, titin molecule, sarcolemma, intercalated disks, and costameres.

**Figure 2:**
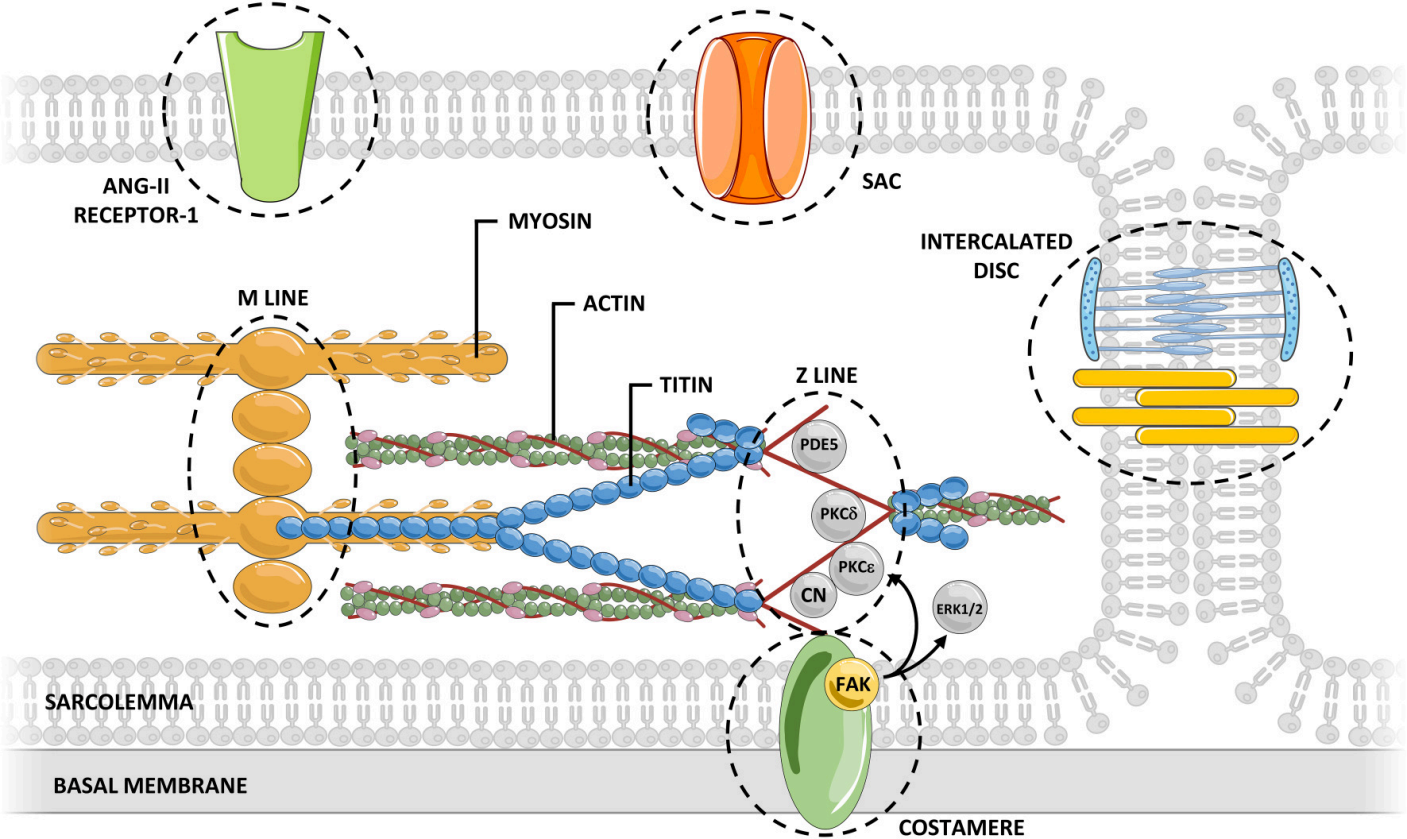
**Myocardial mechanosensing**. Acute myocardial stretch induces an increased myocardial tension that can be sensed by several potential mechanosensors.The connections between mechanosensors and activated signaling pathways are not yet fully understood. CN, calcineurin; ERK, extracellular signal-regulated kinase; FAK, focal adhesion kinase; PDE, phosphodiesterase; PKC, protein kinase C; SAC, stretch-activated channel.

Recent reviews (Takahashi et al., 2013; Buyandelger et al., 2014; Lyon et al., 2015) addressed cardiac mechanotransduction in detail, particularly its relation to remodeling and chronic adaptation. The precise role of mechanosensors in the acute response to stretch is still mostly unknown, as few studies have directly addressed this issue. Therefore, in this topic, we intend to emphasize the great diversity of mechanosensors in the heart and highlight cellular components that, given their structure, mechanism of action and activated signaling pathways, may potentially contribute to the response to acute stretch (Figure 2).

The Z-line divides two adjacent sarcomeres and is traditionally regarded as an essential element to the normal structural arrangement of the sarcomeres. However, its position confers to the Z disk the distinct ability to sense the muscular tension, during either the diastolic or the systolic period (Hoshijima, 2006). The Z-line is composed not only of overlapping actin filaments of adjacent sarcomeres tightly linked by α-actinin, but also of numerous proteins bearing diverse signaling functions (Luther, 2009). Many of these proteins may be relevant for response to acute stretch, including: Protein Kinase C (PKC) ε (Robia et al., 2005), PKCδ (Disatnik et al., 1994), calcineurin (Frey et al., 2000), and phosphodiesterase 5 (Senzaki et al., 2001).

Titin is a giant protein that spans the sarcomere from the Z-line to the M-line. Its molecular structure makes this protein work as a bidirectional spring that determines the myocardial passive tension at different lengths (Castro-Ferreira et al., 2011). In addition to its structural and elastic function, titin also functions as a biomechanical sensor sensing myocardial tension as well as the sarcomeric length. The central role of titin in mechanosensing and its ability to trigger downstream signaling cascades is related to its interactions with numerous structural proteins and signaling proteins at its M-band, Z-line, N2B, N2A, and PEVK-domains (Linke and Krüger, 2010). Titin also features a kinase domain near the carboxyl-terminal (located in the M-line region; Mayans et al., 1998), which may phosphorylate target proteins at specific sites, according to myocardial stretching (Puchner and Gaub, 2010). Moreover, titin presents various phosphorylation sites of its own at PEVK and N2B domains (Krüger and Linke, 2011), whose phosphorylation alters passive tension conferred by titin, either decreasing or increasing it (Ahmed and Lindsey, 2009). This mechanism confers titin a special role in mechanosensing, allowing it to simultaneous function as a mechanosensor and a molecular target. Therefore, it is tempting to hypothesize that upon an increase in acute passive tension, titin is capable of initiating a compensatory mechanism that, ultimately, would lead to decreased titin's passive tension with consequent normalization of sarcomeric passive tension. It is important to highlight that, as the main determinant of myocardial passive tension, modifications of titin phosphorylation status will also modify the activity of remaining mechanosensors.

The sarcolemma separates the intracellular and extracellular environments. This structure is teeming with different receptors subject to activation by extracellular mediators that set different intracellular pathways in motion. However, several sarcolemma proteins can also be activated by cellular stretch, initiating intracellular pathways without binding of extracellular mediators (Storch et al., 2012). Stretch activated channels (SACs) are a paradigmatic example of this type of activation in cytoplasmic membranes (Reed et al., 2014). In cardiomyocytes, upon stretch, SACs are permeable to sodium, potassium and calcium, thereby modulating electrical and mechanical properties of myocardium. The AT1 receptor, a G protein-coupled receptor, can also be directly activated by stretch without the involvement of angiotensin II (Zou et al., 2004). This agonist-independent activation may have a determinant role in the myocardial response to acute stretch. The blockade of this receptor has been associated with a significantly blunted SFR (Heidkamp et al., 2003).

The interaction of cardiomyocytes with neighboring cells (at intercalated discs) and with the extracellular matrix (at costameres) is key to the maintenance of the structural integrity of the myocardium, as well as the transmission of forces between cells (Kresh and Chopra, 2011). Beyond this function, some components of intercalated discs and costameres are known to be involved in mechanosensing (Samarel, 2005). At the level of intercalated discs, the main structures capable of contributing to mechanosensing are fascia adherens junctions (constituted by N-cadherin and associated multi-molecular complex anchoring cytoskeletal actin) and desmosomes (composed of the cell adhesion proteins desmoglein and desmocollin and associated proteins internally linked to the intermediate filament desmin; Sheikh et al., 2009). Both structures are known to contribute to long-term cytoskeletal adaptive responses to different pathophysiological forces (Lyon et al., 2015), but their importance in the acute response remains unknown. Regarding costameres, one important component contributing to mechanosensing are integrins. These heterodimeric transmembrane receptors located at costameres contribute to the connection between extracellular matrix and the intracellular cytoskeleton, and therefore, to the transmission of mechanical signals to the cytoskeleton (Israeli-Rosenberg et al., 2014). One well-known mediator of integrin mechanosensing is focal adhesion kinase (FAK; Domingos et al., 2002b). The signaling resultant of FAK activation is proportional to the magnitude and duration of mechanical stretch (Katz et al., 2002). FAK activity is also related to various signaling pathways potentially relevant to the acute response to stretch, including ERK1/2 (Domingos et al., 2002a) and PKC ε (Heidkamp et al., 2003). Another mediator that may be particularly important for the acute transduction of integrin signaling is integrin-linked kinase (ILK). ILK appears to mediate cardiomyocyte force transduction via regulation of the SERCA activity and phosphorylation of PLB (Traister et al., 2014), which may contribute both to the systolic and diastolic adaptation to acute stretch.

Although several potential mechanosensing molecules have been identified, the connection between these molecules and stretch activated pathways remains elusive. This lack of knowledge is even more pronounced when it comes to the acute activation of these pathways by these molecules. Furthermore, the interrelation between those sensors is complex, and the spread synergism and crosstalk between the pathways activated downstream of those sensors lead to a broad activation of signaling pathways that acutely modulate heart function, which is probably crucial to the ability of myocardium to adapt to acute hemodynamic overload.

### Calcium-dependent pathways

**Figure 3:**
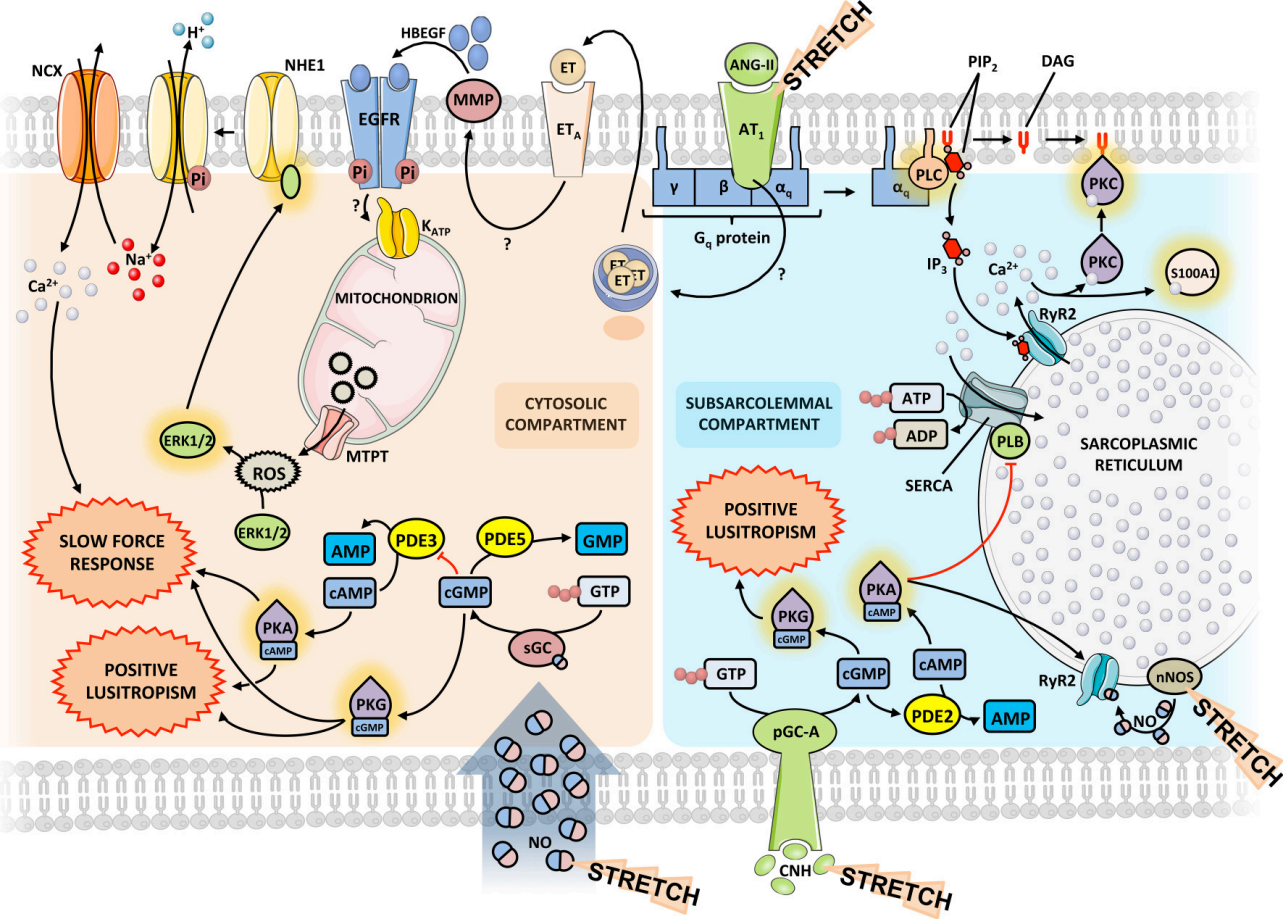
**Integrated signaling pathways**. Diagram representing the molecular interplay between the various players in the acute response to stretch. The same mediators may exert different effects depending on the subcellular compartment. Question marks indicate yet unknown mechanisms. The proposed model is based on data obtained from different species and experimental models. Some signaling pathways may differ between species. ANG-II, angiotensin-II; CNH, cardiac natriuretic hormone; EGFR, epidermal growth factor receptor; ET, endothelin; ERK, extracellular signal-regulated kinase; HBEGF, heparin-binding epidermal growth factor; MMP, matrix metalloproteinase; NCX, sodium-calcium exchanger; NHE, sodium-proton exchanger; nNOS, neuronal NO synthase; PDE, phosphodiesterase; pGC-A, particulate guanylate cyclase A; PK, protein kinase; PLB, phospholamban; PLC, phospholypase C; ROS, reactive oxygen species; Ryr2, ryanodine receptor 2; sGC, soluble guanylate cyclase.

Calcium (Ca^2+^) is the central player in the biphasic systolic adaptation to myocardial stretch. Besides its involvement in the FSM, an increase in the intracellular concentration of Ca^2+^ underlies the SFR (Figure 3). Angiotensin-II (ANG-II) and endothelin (ET) are two peptide hormones (8 and 21 amino acids long, respectively) that share some interesting features. Via binding to receptors coupled to protein G_q_ and subsequent release of Ca^2+^ from intracellular stores and activation of PKC, they increase cardiac inotropism and have a pressor effect on blood vessels (Figure 3). Though they normally play a role in the physiological regulation of blood pressure by altering salt and water balance and vasomotor tone (especially in renal vessels), they have been implicated in the pathophysiology of pulmonary and systemic hypertension and heart failure (Brunner et al., 2006; Mehta and Griendling, 2007).

More recently, the study of the role of local angiotensin and endothelin systems in cardiac function and pathophysiology has been prolific. These have been implicated in the process of cardiac remodeling that takes place as a result of neurohumoral activation in congestive heart failure (Brunner et al., 2006; De Mello and Frohlich, 2011) but they also seem to be central in the modulation of the acute myocardial response to stretch, contributing to the genesis of the FSM and the development of the SFR (Cingolani et al., 2011). In fact, Sadoshima et al. first demonstrated that cultured neonatal cardiomyocytes directly released ANG-II from intracellular vesicles in response to an acute 10 or 30 min stretch, generating a concentration of around 1 nM in the culture medium (Sadoshima et al., 1993). The positive inotropic response to acute stretch is similar to that elicited by addition of exogenous ANG-II in amounts of the same order of magnitude and can be blocked by inhibition of AT_1_ but not AT_2_ receptors (Caldiz et al., 2007). However, this effect seems to depend on downstream release of ET, as inhibition of ET receptors, as well as the ET converting enzyme, ablated both the SFR and the increase in contractility that accompanies ANG-II administration but AT_1_ receptor blocking did not prevent the positive inotropic effect of exogenous ET (Pé́rez et al., 2003). Accordingly, Anderson et al. reported that ET synthesis in myocytes increases following myocardial stretch (Anderson et al., 2004).

Taking these results into account, along with their own, Cingolani's group has since described a complex autocrine/paracrine pathway involving changes in membrane ion currents mediated by ANG-II and ET that sheds some light on the role played by these molecules in the response to myocardial stretch (Cingolani et al., 2011). They propose that the initial step in generating the SFR is an ANG-II dependent release of endogenous ET by poorly described mechanisms that may involve activation of the mineralocorticoid receptor by aldosterone or another non-identified ligand.

Although all three endothelin isoforms have equal positive inotropic potency when exogenously administered, ET-3 is the most likely to be responsible for SFR in a physiological setting (Ennis et al., 2005). The ET receptor then transactivates the epidermal growth factor receptor (EGFR) through various pathways. The best described one involves activation of matrix metalloproteinases and subsequent release of heparin-binding epidermal growth factor (HBEGF), which binds to and activates EGFR. This supposedly triggers an intracellular signaling pathway leading to increased production and release of reactive oxygen species (ROS) by mitochondriae through opening of mitochondrial K_ATP_ channels (Anderson et al., 2004; Villa-Abrille et al., 2010). Redox-sensitive kinases ERK1/2 and p90^rsk^ therefore, increase their activity in response to stretch and phosphorylate the regulatory domain of the sodium-proton exchanger 1 (NHE1), increasing its activity and cytoplasmic [Na^+^]. This transporter has long been implicated in the development of the SFR and this is thought to occur through modulation of ion electrochemical gradients that the increased [Na^+^] provokes. In this setting, the sodium-calcium exchanger (NCX), which usually extrudes calcium, reverses direction of transport, increasing the amplitude of Ca^2+^ transients and, consequently, contractile force (Figure 3).

On the other hand, the increased Ca^2+^ transient, through favored actin-myosin interaction, could result in alarming levels of muscle stiffness. Thus, it is not surprising that other stretch activated mechanisms promote muscle relaxation by improving Ca^2+^ extrusion from cytosol after myocardial contraction. In fact, there is a well-described acute increase in SERCA expression following hemodynamic overload (Kögler et al., 2006). Besides, Ca^2+^ load following stretch binds to the several Ca^2+^ binding proteins in the heart, with S100A1 being the most abundant member of the calcium-binding S100 protein family in myocardial tissue (Duarte-Costa et al., 2014). S100A1-Ca^2+^ complex frees the actin from titin, reducing the passive tension imposed by titin-actin interaction (Yamasaki et al., 2001). The relative importance of these mechanisms in the passive properties of the heart following stretch has not been studied in detail.

The complex loop involving ANG-II and ET still has gaps to be filled and seemingly contradictory results exist in the literature. Our own results confirm the major role of AT_1_ receptors and the NHE-1 and NCX transporters in the development of SFR and the positive inotropic effect of the external addition of ANG-II on myocardium in normal conditions, which subsequently blunts SFR, presumably by saturating the contractile reserve of the pathway in the absence of stretch. In addition, it points to a possible role of PKC in maintaining the SFR during the late phase of acute stretch (Neves et al., 2013).

Interestingly, our group has also described the role of these peptides on the acute modulation of diastolic function through direct effects on both relaxation velocity and myocardial stiffness. Activation of ET_A_ and AT_1_ receptors results in positive lusitropism and decreased stiffness, while ET_B_ and AT_2_ receptors promote negative lusitropism. As in its systolic counterpart, AT_1_ receptor activation seems upstream to endothelin release in the pathway that leads to faster myocardial relaxation, as this effect is dependent on ET_A_ receptor activity and an intact endocardial endothelium (Bras-Silva and Leite-Moreira, 2006; Castro-Chaves et al., 2006). Concurrently, decreasing stiffness depends on the activity of both NHE1 and PKC and on the integrity of the endocardial endothelium (Leite-Moreira et al., 2003, 2006; Bras-Silva and Leite-Moreira, 2006).

### Pathways involving nitric oxide and cardiac natriuretic hormones

Cardiac stretch stimulates both endothelial cells and cardiomyocytes to produce nitric oxide (NO; Casadei and Sears, 2003). In endothelial cells, large amounts of NO are produced by the endothelial nitric oxide synthase (NOS), after which it rapidly diffuses to neighboring cardiomyocytes (Figure 3). In the cardiomyocyte, NO is produced in relatively low levels by both endothelial NOS and neuronal NOS (nNOS; Petroff et al., 2001; Khan et al., 2003).

NO actions can be divided in those that are mediated by an elevation of cGMP (cGMP-dependent effects) and those that do not depend on cGMP elevation (cGMP-independent effects). As NO produced by endothelial cells diffuses through the cardiomyocyte, soluble guanylate cyclase present in the cytoplasm is activated, leading to an increase in cGMP concentration in the cytosolic compartment. The effect of cGMP is mediated through protein kinase G (PKG), which is able to phosphorylate TnI, rendering a reduction of myofilamentary sensitivity to calcium, therefore increasing the rate of Ca^2+^ dissociation (Layland et al., 2005). NO produced by nNOS, which is located in the sarcoplasmic reticulum membrane, simultaneously modulates the diastolic properties of the heart through PKA-dependent phosphorylation of PLB in a cGMP-independent pathway (Zhang et al., 2008). The phosphorylation of PLB disinhibits SERCA, which rapidly reuptakes free cytoplasmic Ca^2+^. Taken together, NO mediated pathways, both cGMP-dependent and cGMP-independent pathways, hasten myocardial relaxation (positive lusitropism), prolonging the diastolic time interval while reducing the passive tension of cardiomyocytes. These changes in myocardial properties are crucial for the heart to accommodate increasing preloads, without substantial changes in the intracardiac filling pressures (Figure 3).

Besides altering the diastolic properties of the heart, NO also modulates its contractility. NO-derived cGMP coexists in the cytoplasm with several members of the phosphodiesterase (PDE) family. Proteins of this family are metallophosphohydrolases that cleave cGMP and/or cAMP to 5′-GMP and 5′-AMP, inhibiting the activation of PKG and Protein Kinase A (PKA), respectively (Zaccolo and Movsesian, 2007). Therefore, although stretch induces an increase in the production of NO, the cytoplasmic concentration of NO-derived cGMP is kept relatively low due to continuous hydrolysis mediated by PDE5, located preferentially in Z-bands, in a cGMP-activated negative feedback mechanism. An important crosstalk between cGMP and cAMP pathways occurs, as low concentrations of cGMP inhibit PDE3, thus preventing the hydrolysis of cAMP (Francis et al., 2010). Accumulated cytoplasmic cAMP activates PKA, which is then responsible for the phosphorylation of several proteins involved in cardiac contraction (Figure 3). These include the sarcolemmal voltage-gated L-Type Ca^2+^ channels, RyR2, PLB, the phosphatase 1 inhibitor, TnI and MyBP-C, all aiming to increase myocardial contractility (Fischmeister et al., 2006). Interestingly, a prolonged activation of RyR2 could ultimately lead to SR Ca^2+^ depletion, and consequently to a paradoxal negative inotropism; however, activation of SERCA, as previously stated, is able to replenish the SR Ca^2+^ pool and maintain this cycle. RyR2 can also be opened via cyclic adenosine diphosphate ribose, which is produced by a PKG mediated phosphorylation of adenosine diphosphate ribosylcyclase (Willmott et al., 1996). NO can also exert an important effect on the systolic properties of the myocardium *per se* in a cGMP-independent pathway. NO produced by nNOS directly S-nitrosylates reactive thiol residues within RyR2, which also contributes to an increased Ca^2+^ release (Wang et al., 2010). Taken together, NO mediated pathways, both cGMP-dependent and cGMP-independent pathways, enhance myocardial contractility (positive inotropism) in response to stretch, allowing the heart to pump more vigorously the increasing blood volume arriving after each heartbeat (Figure 3). Accordingly, we recently demonstrated in rabbit papillary muscles that acute myocardial stretch in the presence of a PKG inhibitor leads to a significant attenuation of the SFR (Castro-Ferreira et al., 2014).

Cardiac stretch also stimulates cardiomyocytes to release cardiac natriuretic hormones (CNH), namely atrial natriuretic peptide and brain natriuretic peptide. Both exert their cardiac effect by activating cell surface-associated particulate guanylate cyclase A, which in turn increases the concentration of cGMP in the subsarcolemmal compartment (Francis, 2010; Figure 3). However, in opposition to NO-derived cGMP, whose concentration is kept low through a PDE5 mediated negative feedback mechanism, CNH-derived cGMP triggers a feed-forward mechanism that increases cGMP concentration even more (Castro et al., 2010). While this important difference is not reflected in the relaxation properties of the myocardium, as similarly to NO, CNH exert a positive lusitropic effect through phosphorylation of PLB and TnI, high levels of cGMP stimulate PDE2, reducing cAMP levels, which explains the absence of a positive inotropic effect following CNH release (Potter et al., 2006).

Both NO and CNH can exert their effects through cGMP; however, their physiological role may be quite different. The production/degradation of NO is finely tuned and occurs at a very high pace, meaning that NO must probably be related to adaptations to acute stretch on a beat-to-beat basis (e.g., in inspiration, changing from orthostatic to lying position). Contrastingly, and taking into account their diuretic effects in the kidney and longer half-life than NO, CNH could be more important in hypervolemic, hypertensive states, where stretch is prolonged in time, which explains the importance in relaxing the myocardium without increasing contractility, which would be deleterious.

### Effects of ischemia on the myocardial response to acute stretch

Myocardial ischemia is a paradigmatic situation of acute hemodynamic overload in which abnormal myocardial loading activates a variety of cellular responses. During an ischemic event, several neurohumoral agents are released, which contribute to the overall physiologic process of cardiac adaptation to hemodynamic overload. Though significant advances in knowledge have been made in the last three decades regarding the processes involved in the pathophysiological consequences of ischemia *per se*, the cardiac response to stretch under those conditions is just taking its first steps.

As previously stated, the myocardial response to stretch is a two-step adaptive response. The contractile response following acute stretch under ischemic conditions has been less studied. In a recent study carried out by our group, it was clearly demonstrated that this response is profoundly affected, as shown not only by a blunted FSM but also by the abolishment of the SFR (Neves et al., 2013), thus supporting some previous indirect observations (Goto et al., 1988; Owen et al., 1993). In the human heart, after an occlusion of a coronary artery, pH falls within 15 s (Poole-Wilson, 1989), possibly contributing to the loss of contractility (Steenbergen et al., 1977; Patangay et al., 2009; Decker et al., 2012) due to a reduced sensitivity of the myofilaments to calcium (Kihara et al., 1989; Salas et al., 2006), consequently leading to a diminished Ca^2+^ availability to bind to troponin C. Metabolic abnormalities should not be excluded, as they lead to insufficient energy supply to support cardiac work due to MgADP accumulation, which decreases the sliding velocity in the vicinity ATPase, slowing down the contraction cycle (Sata et al., 1996) and impairing the FSM (Robinson et al., 2002). These mechanisms are probably responsible for the observed ablation of the SFR in these conditions. A possible explanation for this was given recently by our group, demonstrating that this effect was AT1R-dependent and partially AT2R-dependent: while in the absence of blockade of angiotensin II receptors the myocardium presents a progressive decrease in contractility during ischemia, the blockade of AT1R is able to prevent this deterioration after stretch (Neves et al., 2013). This effect may also be mediated by AT2R activation through bradykinin, PKC, and cGMP (Jugdutt and Balghith, 2001).

Little is known about the role of the PKC under ischemic conditions. As previously stated, PKC activity is more significant during the late phase of the SFR, preventing the development of a slow force decline. Also, under ischemic conditions, PKC was not capable of modifying contractile performance (Neves et al., 2013). This is consistent with its inability to alter the systolic adaptation during early phase of the response to stretch and does not exclude that its downstream mediators may be compromised in such conditions. However, PKC has several isoforms, some of them present within the same cell and activated by the same stimuli but showing different and, at times, opposite effects. For example, in ischemic heart disease, PKCδ induced decreased ATP generation (Inagaki et al., 2003), and therefore, insufficient energy supply may play a role. As such, it would be interesting to study how each one modulates the contractile response to stretch under ischemic conditions.

Usually, in the setting of myocardial infarction, the PKG signaling pathway through NO and CNH is modulated pharmacologically by nitrates as NO donors. Also, administration of PDE5 inhibitors may be promising therapeutic targets (Kukreja, 2013). However, only recently the specific role of the PKG signaling pathway in ischemic conditions was shown. Intriguingly, activation of PKG pathway did not alter the SFR in ischemic conditions, while PDE5 inhibition significantly mitigated the contractile decline after stretch in ischemia (Castro-Ferreira et al., 2014). This can be explained by NO/cGMP-independent cardioprotective effects (Elrod et al., 2007). However, the mechanisms responsible for the protective action of the sildenafil remain to be fully elucidated.

### Diastolic response to acute myocardial stretch

Contrarily to the systolic adaptation (composed by the well-characterized FSM and SFR), the existence of a diastolic response to acute stretch remains unknown, as it has not been directly evaluated by any study. However, indirect evidence suggests that a diastolic response to acute myocardial is highly probable. First, the main determinant of myocardial stiffness, titin, can be acutely phosphorylated by several signaling pathways that are activated upon stretch, including calcium-dependent pathways, and pathways involving NO and CNH (Ladeiras-Lopes et al., 2009). Furthermore, the phosphorylation of titin by those pathways is known to induce important changes in diastolic cardiomyocyte properties that may be of physiological relevance (Castro-Ferreira et al., 2011). On the other hand, most of the sarcomeric proteins and ion channels involved in the SFR, are also known to have an impact in diastolic function: for example, the increased phosphorylation of TnI and of MyBPC are known to improve relaxation (Layland et al., 2005; Mamidi et al., 2014). Similarly, the increased activity of SERCA after myocardial stretch probably contributes to a decreased concentration of diastolic cytosolic free Ca^2+^ and, thus to a decreased stiffness dependent of myosin-actin interaction during the diastolic period (Ladeiras-Lopes et al., 2009).

The relative importance of this potential mechanism of diastolic adaptation in comparison to the systolic adaptation is obviously also unknown. The most probable is that the diastolic adaptation interacts with the systolic adaptation to allow a complete response to hemodynamic challenges. It is tempting to hypothesize that a decreased diastolic reserve and an inability to develop a diastolic response to acute stretch contributes to the pathophysiology of heart failure with preserved ejection fraction. Those patients are known to have a low exercise capacity, a poor tolerance to volume overload, and a high risk of acute pulmonary edema (Aurigemma and Gaasch, 2004; Maeder and Kaye, 2009), all potentially explained by a failure of the left ventricle to improve its diastolic function in response to a hemodynamic overload leading to an excessive end diastolic pressure and consequently to pulmonary congestion. The need to design studies to directly evaluate the diastolic response to acute stretch is in our opinion a priority in this field, as it may unravel a completely new pathway of research.

## Conclusion

The myocardial response to acute stretch represents a fundamental adaptive capacity of the heart. Although this functional response to hemodynamic overload has been known for long time, the structures responsible for mechanosensing, as well as the pathways involved in the response, are still only partially clarified. The possibility of concomitant diastolic adaptation, although theoretically appropriate, has never been directly assessed.

Despite the relevant knowledge accumulated on the cardiac response to acute hemodynamic overload, there is much that we still do not know. The quest for further understanding of the mechanosensing involved in this mechanism, the pathways responsible for the response and the characterization of the diastolic adaptation may provide new insights, not only in its physiological importance, but also in the prevention and treatment of cardiovascular diseases, such as heart failure with reduced ejection fraction, heart failure with preserved ejection fraction, hypertensive crises or ischemic heart disease.

## Author contributions

JSN, AML-M, MN-S, JA-C, and RC-F contributed to the conception and design of the work, reviewed the bibliography, drafted the work, revised the work, and approved the final version. AFL-M contributed to the conception and design of the work, critically revised the work and approved the final version.

## Funding

This work was supported by grants from the Portuguese Foundation for Science and Technology (EXCL/BIM-MEC/0055/2012), the Cardiovascular R&D Unit (UIC/IC/00051/2013), and from the European Commission (FP7-Health-2010; MEDIA-261409).

### Conflict of interest statement

The authors declare that the research was conducted in the absence of any commercial or financial relationships that could be construed as a potential conflict of interest.
